# Permissive Weight Bearing in Patients With Surgically Treated Periprosthetic Femoral Fractures Around Total Hip Arthroplasty: A Scoping Review

**DOI:** 10.7759/cureus.56374

**Published:** 2024-03-18

**Authors:** Maud AM Vesseur, Bob Heijkens, Jetse Jelsma, Yoeri FL Bemelmans, Marion JLF Heymans, Raoul Van Vugt, Bert Boonen, Martijn GM Schotanus

**Affiliations:** 1 Department of Orthopedic Surgery, Zuyderland Medical Center, Heerlen, NLD; 2 Department of Orthopedic Surgery, Sint Maartenskliniek, Ubbergen, NLD; 3 Department of Quality Improvement, Zuyderland Medical Center, Heerlen, NLD; 4 Department of Science, Zuyderland Medical Center, Heerlen, NLD; 5 Department of Surgery, Zuyderland Medical Center, Heerlen, NLD; 6 Department of Epidemiology and Public Health, Maastricht University Care and Public Health Research Institute, Maastricht, NLD

**Keywords:** permissive weight bearing, weight-bearing protocols, surgery, trauma, ppff, periprosthetic femoral fractures, pwb

## Abstract

Periprosthetic femoral fractures (PPFF) around total hip arthroplasty (THA) are one of the leading causes of hip revision. High mortality rates are observed after revision in case of PPFF around THA. To modify risk factors, early postoperative mobilization is necessary. Permissive weight bearing (PWB) is designed to optimize clinical recovery in aftercare. This study aimed to perform a scoping review to summarize the current available evidence on postoperative weight bearing in late PPFF around THA and the implementation of PWB in aftercare. A systematic search was performed on the Cochrane Library, Web of Science, Ovid MEDLINE, EMBASE, and CINAHL databases on January 26th, 2023. Articles were screened in two stages by two independent reviewers. Studies describing adult patients with a history of primary THA who were surgically treated for late PPFF and mentioning prescribed postoperative weight-bearing protocols with relevant outcome measures were included. Seven studies were included, reporting data on 22 patients (age range 47-97 years, BMI range 19-32 kg/m^2^, ASA classification range 2-3). No studies used PWB in aftercare. The non-weight-bearing group showed no complications. The restricted weight-bearing group had one death and one implant failure. The full weight-bearing group experienced one deep infection and one plate removal because of impingement. The main finding was that, after an extensive systematic search, no articles could be included focusing on PWB in patients with a late PPFF after THA. Addressing this gap in the literature is essential to advancing the understanding of postoperative weight-bearing protocols and PWB for late PPFF around THA.

## Introduction and background

The incidence of primary total hip arthroplasty (THA) is rising, and subsequently, more periprosthetic femoral fractures (PPFF) around THA occur [1]. These PPFFs can occur intra- or postoperatively [2]. A late PPFF is considered a PPFF that occurs after new trauma, and therefore, it does not refer to a PPFF that occurred during the operation or in the immediate days following index surgery without new trauma. The incidence of late PPFF ranges between 0.07% and 3.5% [3,4]. The average mean age at which late PPFF is reported in the existing literature is within the range of 60-77 years [5]. PPFF is one of the leading causes of THA revision, following mechanical implant failure, metallosis, and implant instability [6]. Higher mortality rates are observed after THA revision in the case of a PPFF: one in 13 patients is deceased within two years after surgery compared to revision based on infection or recurrent dislocation (both one in 20 patients) [7]. This is significantly higher compared to the overall mortality rate of one in 51 patients [7]. Decreased mobility leads to a higher risk of pneumonia and delirium and subsequently increases mortality rates [8,9].

With the use of early postoperative mobilization, risk factors leading to higher postoperative mortality rates can be reduced [8,9]. Potential risk factors should be addressed as soon as PPFF is diagnosed. This apparent vulnerable patient group is in need of early and pain-free mobilization. To achieve these treatment goals and to optimize rapid clinical recovery and restoration of function, permissive weight bearing (PWB) has been introduced as a relatively new aftercare mobilization regimen [10]. The initial studies on PWB in different types of lower extremity fractures have shown successful outcomes without raising the number of complications [10]. PWB enables patients to mobilize earlier compared to patients who follow a non-weight-bearing protocol, aiming for a lower risk of mortality [11]. However, PWB is not a commonly used postoperative weight-bearing protocol, and there is no consensus on its use in the aftercare of PPFF around THA [12].

The term PWB was first described by Kalmet et al. [13] and is now increasingly described in the literature and applied in clinical postoperative weight-bearing treatment protocols [14]. PWB involves advancing the weight-bearing process based on the patient's and therapist's subjective observations, such as pain levels and the patient's ability to tolerate weight-bearing, while also taking into account objective factors like limb temperature, swelling, and gait metrics [13].

This study aims to perform a scoping review, with the use of a systematic search, to summarize the current available evidence on postoperative weight bearing in late PPFF around THA and the implementation of PWB in aftercare regarding PPFF in THA patients.

## Review

Material and methods

The Preferred Reporting Items for Systematic Reviews and Meta-Analyses extension for Scoping Reviews (PRISMA-ScR) checklist was used to draft the protocol and to perform this review [15]. The study protocol was registered in the Prospero database with registration number CRD42021230271.

Selection Criteria

The suitability of articles was assessed in two stages. Inclusion criteria were adult patients (age >18 years) with a history of primary THA with a surgically treated late PPFF (Vancouver classification A, B, or C). Articles were suitable for inclusion when describing postoperative weight-bearing protocols with relevant outcome measures (time of hospital stay, resuming activities of daily living, time to fracture union, time to first mobilization, time to full weight bearing, range of motion, quality of life, pain, postoperative complications, readmission, and mortality rate). Studies were included in different groups based on the postoperative weight-bearing protocols. Non-weight bearing (NWB) group; restricted weight bearing (RWB) group, which also includes partial weight bearing and toe touch weight bearing; permissive weight bearing (PWB) group, also including weight bearing as tolerated; and full weight bearing (FWB) group, encompassing also immediate weight bearing. Exclusion criteria were animal studies, editorial articles, and abstracts of congresses. Studies reported in another language than English or Dutch were also excluded. There were no restrictions on study quality, year of publication, or study origin.

Search and Screening Process

An extensive systematic literature search was conducted by two reviewers (MV and MH) on January 26th, 2023, using the Cochrane Library, Web of Science, Ovid MEDLINE, EMBASE, and CINAHL. The search strategy was reviewed by the other reviewers in this study. No search limitations or filters were used. Search terms are presented in Tables 1-6.

**Table 1:** Cochrane Library ti.ab.kw: title word, abstract word, keywords (MeSH and other), and word variations have been searched

#5	#2 AND #4	442
#4	#1 OR #3	10,501
#3	((fracture* AND (periprosthetic* OR 'peri implant*' OR femoral OR femur OR 'periprosthetic femoral' OR 'periprosthetic femur' OR hip OR trochanteric OR intertrochanteric OR subtrochanteric OR 'peri prosthetic*' OR 'peri prosthetic femoral' OR 'peri prosthetic femur' OR 'femur torsion'))):ti,ab,kw	9,533
#2	('weight bearing' OR weightbearing OR loadbearing OR 'load bearing' OR 'axial load*' OR pwb OR 'permissive weight*' OR 'permissive load' OR 'load carry*'):ti,ab,kw	6,064
#1	('revision arthroplast*'):ti,ab,kw	1,147
	Total	442
	Cochrane Reviews	6
	Trials	436

**Table 2:** Web of Science TS: topic; searched for topic terms in the following fields within a record: title, abstract, author keywords, and Keywords Plus®

#5	#3 AND #4	3,001
#4	TS=("weight bearing" OR weightbearing OR loadbearing OR "load bearing" OR "axial load*" OR pwb OR "permissive weight*" OR "permissive load" OR "load carry*")	66,576
#3	#1 OR #2	96,048
#2	TS=(revision arthroplast*)	22,379
#1	TS=(((fracture* AND (periprosthetic* OR "peri implant*" OR femoral OR femur OR "periprosthetic femoral" OR "periprosthetic femur" OR hip OR trochanteric OR intertrochanteric OR subtrochanteric OR "peri prosthetic*" OR "peri prosthetic femoral" OR "peri prosthetic femur" OR "femur torsion") ))) AND TS=(((fracture* AND (periprosthetic* OR "peri implant*" OR femoral OR femur OR "periprosthetic femoral" OR "periprosthetic femur" OR hip OR trochanteric OR intertrochanteric OR subtrochanteric OR "peri prosthetic*" OR "peri prosthetic femoral" OR "peri prosthetic femur" OR "femur torsion") )))	77,850

**Table 3:** Ovid MEDLINE TS: mp.: title, book title, abstract, original title, name of substance word, subject heading word, floating sub-heading word, keyword heading word, organism supplementary concept word, protocol supplementary concept word, rare disease supplementary concept word, unique identifier, synonyms. exp: “explodes” controlled vocabulary term (e.g., expands the search to all more specific related terms in the vocabulary’s hierarchy)

#10	#6 AND #9	3,163
#9	#7 OR #8	44,103
#8	(weight bearing or weightbearing or loadbearing or load bearing or axial load* or pwb or permissive weight* or permissive load or load carry*).mp.	44,103
#7	exp Weight-Bearing/	21,767
#6	#1 OR #2 OR #3 OR #4 OR #5	66,138
#5	(Fracture* and (Periprosthetic* or Peri Implant* or femoral or femur of periprosthetic femoral or periprosthetic femur or Trochanteric or Intertrochanteric or Subtrochanteric or peri prosthetic* or peri prosthetic femoral or peri prosthetic femur or femur torsion)).mp.	51,713
#4	exp Periprosthetic Fractures/	1,541
#3	exp Hip Fractures/	28,172
#2	exp Femoral Fractures/	44,387
#1	revision arthroplasty.mp.	2,268

**Table 4:** EMBASE exp: “explodes” controlled vocabulary term (e.g., expands the search to all more specific related terms in the vocabulary’s hierarchy). ti,ab,kw: title, abstract, and authentication keywords

#9	#5 AND #8	5,304
#8	#6 OR #7	51,589
#7	‘weight bearing':ti,ab,kw OR weightbearing:ti,ab,kw OR loadbearing:ti,ab,kw OR 'load bearing':ti,ab,kw OR 'axial load':ti,ab,kw OR pwb:ti,ab,kw OR 'permissive weight*':ti,ab,kw OR 'permissive load':ti,ab,kw OR 'load carry':ti,ab,kw	32,559
#6	‘weight bearing’/exp	33,622
#5	#1 OR #2 OR #3 OR #4	109,702
#4	Fracture:ti,ab,kw AND (periprosthetic*:ti,ab,kw OR 'peri implant*':ti,ab,kw OR femoral:ti,ab,kw OR femur:ti,ab,kw OR 'periprosthetic femoral':ti,ab,kw OR 'periprosthetic femur':ti,ab,kw OR hip:ti,ab,kw OR trochanteric:ti,ab,kw OR intertrochanteric:ti,ab,kw OR subtrochanteric:ti,ab,kw OR 'peri prosthetic*':ti,ab,kw OR 'peri prosthetic femoral':ti,ab,kw OR 'peri prosthetic femur':ti,ab,kw OR 'femur torsion':ti,ab,kw) OR 'revision arthroplasty*':ti,ab,kw	76,835
#3	‘femur fracture’/exp	46,153
#2	‘hip fracture’/exp	56,158
#1	'periprosthetic fracture'/exp	4,216

**Table 5:** CINAHL MH: search the exact CINAHL® subject heading and search both major and minor headings, TI: title, AB: abstract

#9	#5 AND #8	808
#8	#6 OR #7	14,447
#7	TI ( ((“Weight Bearing” OR Weightbearing OR Loadbearing OR “Load Bearing” OR “Axial Load*” OR PWB OR “permissive weight*” OR “permissive load” OR “load carry*”)) ) OR AB ( ((“Weight Bearing” OR Weightbearing OR Loadbearing OR “Load Bearing” OR “Axial Load*” OR PWB OR “permissive weight*” OR “permissive load” OR “load carry*”)) )	8,934
#6	MH weight-bearing	8,258
#5	#1 OR #2 OR #3 OR #4	23,128
#4	TI ( (("Periprosthetic Fracture*" OR "Peri Implant Fracture*" OR "femoral fracture*" OR "femur fracture*" OR "periprosthetic femoral fracture*" OR "periprosthetic femur fracture*" OR "Hip fracture*" OR "Trochanteric Fracture*" OR "Intertrochanteric Fracture*" OR "Subtrochanteric Fracture*" OR "fracture of the hip*" OR "revision arthroplast*" OR "peri prosthetic fracture*" OR "peri prosthetic femoral fracture*" OR peri prosthetic femur fracture* OR femur torsion fracture*)) ) OR AB ( (("Periprosthetic Fracture*" OR "Peri Implant Fracture*" OR "femoral fracture*" OR "femur fracture*" OR "periprosthetic femoral fracture*" OR "periprosthetic femur fracture*" OR "Hip fracture*" OR "Trochanteric Fracture*" OR "Intertrochanteric Fracture*" OR "Subtrochanteric Fracture*" OR "fracture of the hip*" OR "revision arthroplast*" OR "peri prosthetic fracture*" OR "peri prosthetic femoral fracture*" OR peri prosthetic femur fracture* OR femur torsion fracture*)) )	16,744
#3	MH periprosthetic fractures	322
#2	MH femoral fractures	5,658
#1	MH hip fractures	12,380

**Table 6:** Summary

1	Cochrane library	442
2	Web of Science	3,001
3	Ovid MEDLINE	3,163
4	EMBASE	5,304
5	CINAHL	808
	Total	12,718

**Figure 1:**
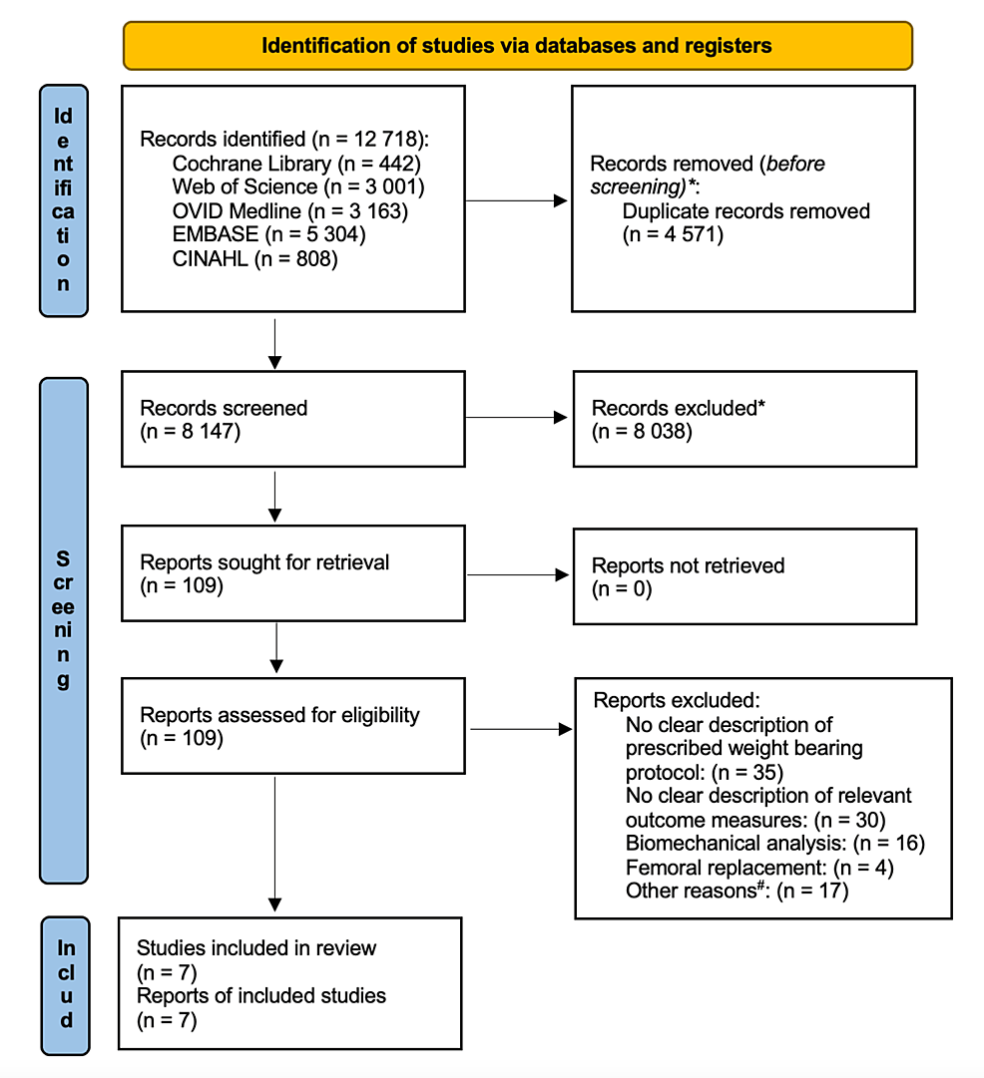
PRISMA flowchart * Without the use of automation tools or artificial intelligence, # Other reasons: neglected femoral neck fracture of the other side wherefore ambulated with partial weight bearing (n=1), atypical femoral fractures because of long-time bisphosphonate use (n=4), implant failure (n=7), open PPFF (n=1), bilateral Vancouver A PPFF within short postoperative time wherefore multiple operations followed (n=1), in combination with uncontrolled infection (n=1) and combination with acetabulum fracture (n=2) PRISMA: Preferred Reporting Items for Systematic Reviews and Meta-Analyses, PPFF: periprosthetic femoral fractures Image Credit: Page et al. (2021) [16]

The results of the literature search were collected in a reference management program (RefWorks, ProQuest LLC). All exact duplicates were marked and deleted until one original article remained. All close duplicates were marked and screened by one reviewer (MV); if these appeared to be exact duplicates, they were also deleted until one original article remained. First, all articles were screened on title and abstract (TiAb) independently by two reviewers (MV and BH). Studies that did not meet the inclusion criteria were excluded. Second, the remaining articles went through a second independent selection for full-text screening by the same two reviewers. No automation or artificial intelligence tools were used for article selection and/or inclusion. Any disagreement between the two reviewers was resolved by a third reviewer (MS). When a systematic review was identified, it was screened for missing studies in the present initial search, and if present, they were included in the present review. A flowchart of the selection process is presented in Figure 1.

Data Collection

A data-charting form was developed by two reviewers (MV and BB) to determine which variables to extract. One reviewer (MV) independently extracted data from the included studies. The extracted data was put in an Excel charting form (Microsoft Excel, Version 6.77, 2023 Microsoft). The following study characteristics were collected if present: study ID (author, year), country, study design, number of patients (n), age in years (mean), sex (female, male), medical morbidities (e.g., American Society of Anesthesiologists (ASA) physical status classification, body mass index (BMI), smoking), fracture classification (Vancouver classification A, B, or C), surgical intervention details, postoperative weight-bearing protocol, and earlier described relevant outcome measures. This was checked by all the other reviewers. The final version of the charting form is available in Table 7.

**Table 7:** Final version of the charting form

Author	Year of publication	Origin/country of origin	Study design	Study population	Medical morbidities	Type of fracture	Surgical intervention details	Weight-bearing protocol	Outcomes
First author of the paper included	The year that the paper was first published	The country where the study was originally conducted	The study design which was used in the study	Relevant characteristics of the study population	Relevant comorbidities for the included patients	Type of PPFF with the use of the Vancouver classification	Details on the operative technique used to treat the mentioned PPFF	Clear description of the prescribed postoperative weight-bearing protocol	Relevant outcome measures mentioned in the paper

Results

Search results based on the number of studies are presented in Tables 1-6. The search resulted in a total of 12,718 records, and 4,571 records were removed before screening based on duplicates. Eight thousand one hundred forty-seven (8,147) records were screened on TiAb, and 8,038 records were excluded based on the inclusion and exclusion criteria. The remaining 109 reports were assessed for eligibility. Based on full-text screening, 102 reports were excluded for the following reasons: no clear description of the prescribed weight-bearing protocol (n=35), no clear description of relevant outcome measures (n=30), biomechanical analysis (n=16), femoral replacement (n=4), and other reasons (n=17). This left seven primary studies including 22 patients (age range 47-97 years, BMI range 19-32 kg/m2, ASA classification range 2-3) between 2006 and 2021 for final inclusion [17-23]. The flowchart of the screening process is presented in Figure 1.

A study by Martinov et al. [17] was included in two different postoperative weight-bearing protocol groups because of the different management of postoperative weight-bearing for each specific patient. One study reported outcome data on the treatment of late PPFF within the NWB group [17]. Six studies adhered to the RWB group [17-22]. No studies were included in the PWB group. One study presented results after surgical treatment of late PPFF in the FWB group [23]. No studies reported outcomes on Vancouver type A (OTA 32A1[IV.3A12] [24]), four studies on Vancouver type B1 (OTA 32A1[IV.3B1] [24]) [19-21,23], two studies on Vancouver type B2 (OTA 32A1[IV.3B2] [24]) [17,22], one study on Vancouver type B3 (OTA 32A1[IV.3B3] [24]) [18], and one study on Vancouver type C (OTA 32A1[IV.3C] [24]) fractures [23].

Open reduction and internal fixation (ORIF) was the most commonly used technique in the included studies [17-21,23] with the use of different postoperative weight-bearing regimes, including patients in the NWB group [17], RWB group [17-21], and FWB group [23]. This surgical technique led to one deep infection in the FWB group (time frame not mentioned) [23], one death at a two-month follow-up in the RWB group [20], one plate fracture at a 12-month follow-up needing revision in the RWB group [21], and one plate removal due to impingement in the FWB group (time frame not mentioned) [23]. One stem revision was done in the RWB group without any complications mentioned [22].

More detailed information on study characteristics of interest and relevant outcome measures is summarized in Table 8.

**Table 8:** Overview of the included studies yo: years old, BMI: body mass index, ASA: American Society of Anesthesiologists physical status classification, FJS: Forgotten joint score, OHS: Oxford hip score, ORIF: open reduction and internal fixation, MIPO: minimally invasive plate osteosynthesis, kg: kilogram, LCP: locking compression plate, LISS: less-invasive stabilization system, FWB: full weight bearing, NWB: non-weight bearing, RWB: restricted weight bearing, PWB: permissive weight bearing

Author	Year of publication	Origin/country of origin	Study design	Study population	Medical morbidities	Type of fracture	Surgical intervention details	Weight-bearing protocol	Outcomes
NWB group									
Martinov et al. [17]	2021	Belgium and Canada	Retrospective study design	One patient; 84yo female	BMI of 19 and ASA 2	Vancouver B2	Hook plate with screws and cerclage wires	Weight-bearing was not allowed for 12 weeks after surgery	Hospital stay of six days; at the final follow-up at three years, there were no complications besides subsidence and varus migration of the stem and the proximal cerclage wires cut through the medial cortex; FJS of 72; and OHS of 32.
RWB group									
Martinov et al. [17]	2021	Belgium and Canada	Retrospective study design	Two patients: Patient 1; 70yo male. Patient 2; 94yo female	Patient 1; BMI of 32 and ASA 3. Patient 2; BMI of 22 and ASA 3	Patient 1: Vancouver B2. Patient 2: Vancouver B2	Patient 1: ORIF with two plates and cerclage wires. Patient 2: hook plate with screws and cerclage wires	Patient 1: eight weeks partial weight bearing. Patient 2: toe-touch walking for six weeks	Patient 1: hospital stay of four days; at final follow-up at 4.5 years, there were no complications; FJS of 92; and OHS of 42. Patient 2: hospital stay of seven days; at final follow-up at five years, there were no complications besides slight radiolucency of the lateral cortex and greater trochanter; FJS of 82 and OHS of 34; and she was walking without a walking aid.
Matar et al. [18]	2021	United Kingdom	Retrospective study design	Two patients: Patient 1; 89yo female. Patient 2: 68yo female	Not mentioned	Vancouver B3	Patient 1: ORIF with plate and strut anterior. Patient 2: ORIF with plate and strut anterolateral	Protected weight bearing for six weeks	Patient 1: time to union: six months; at final follow-up at 4.2 years, there were no complications. Patient 2: time to union: five months; at the final follow-up at four years, there were no complications
White [19]	2013	United States of America	Case report	One patient; 84yo female	Dementia, cardiovascular disease, atrial fibrillation	Vancouver B1	ORIF with plate and screw fixation	Allowed protected weight bearing and FWB at eight weeks	First mobilization on day 1. At three months, the patient was able to walk without pain, and X-rays at eight months showed solid union
Apivatthakakul et al. [19]	2012	Thailand	Retrospective study design	Seven patients: Patient 1; 84yo female. Patient 2: 74yo male. Patient 3: 47yo male. Patient 4: 80yo female. Patient 5: 79yo female. Patient 6: 80yo female. Patient 7: 78yo female	Not mentioned	Vancouver B1	Closed percutaneous cerclage wiring with internal fixation with MIPO utilizing a long LCP Synthes bridging plate	Partial weight bearing of 10-15 kg in the first week. Afterwards, gradually weight bearing as tolerated	Patient 1: time to union: 16 weeks; at the final follow-up at 15 months, there were no complications. Patient 2: time to union: 20 weeks; at the final follow-up at 12 months, there were no complications. Patient 3: time to union: 20 weeks; at final follow-up at 18 months, there were no complications. Patient 4: time to union: 18 weeks; at final follow-up at 12 months, there were no complications. Patient 5: time to union: 20 weeks; at the final follow-up at 12 months, there were no complications. Patient 6 died two months after the operation due to cardiovascular problems. Patient 7: time to union: 18 weeks; at final follow-up at 12 months, there were no complications
Buttaro et al. [21]	2007	Argentina	Retrospective study design	Three patients: Patient 1: 85yo male. Patient 2: 80yo female. Patient 3: 88yo male	Not mentioned	Vancouver B1	LCP fixation	Early mobilization, walking with two crutches or a walker, and toe-touch weight bearing on the involved side for 45 days. In the absence of pain and radiographic findings, patients progressed to 20% weight bearing until the ninetieth day. Afterward, gradually, weight bearing was tolerated, and a cane was used.	Patient 1: time to union at six months; plate pullout at six months; at final follow-up at 20 months, there were no complications. Patient 2: time to union 12 months after re-operation; plate fracture at 12 months; revision with locking compression plate and struts; at the final follow-up at 12 months, there were no further complications. Patient 3: time to union: six months; at final follow-up at 12 months, there were no complications .
Ferrara et al. [21]	2006	United States of America	Case report	One patient; 78yo male	Not mentioned	Vancouver B2	Fully coated femoral stem revision implant	Toe touch weight bearing on the affected extremity, at two months advanced to weight bearing as tolerated	Fracture union was achieved without any complications. The time frame or final follow-up period was not mentioned.
PWB group									
.	.	.	.	.	.	.	.	.	.
FWB group									
Anakwe et al. [23]	2008	United Kingdom	Retrospective study design	Five patients: Patient 1: 72yo male. Patient 2: 77yo female. Patient 3: 89yo female. Patient 4: 81yo female. Patient 5: 97yo male	Patient four had medical treatment for osteoporosis	Patient 1: Vancouver B1. Patient 2: Vancouver C. Patient 3: Vancouver B1. Patient 4: Vancouver C. Patient 5: Vancouver B1	LISS plate fixation	Mobilize touch weight bearing initially in the postoperative phase. In practice, many of these elderly patients found this difficult, and FWB was accepted	Patient 1: time to union: 28 weeks; regained pre-injury mobility; no complications. Patient 2: time to union: 16 weeks; went from stick mobilization to hoist mobilization; had a plate removed because of knee pain or impingement. Patient 3: time to union: 26 weeks; went from independent mobilization to in-house mobilization; had a plate removed because of a deep infection. Patient 4: time to union: 18 weeks; went from independent mobilization to stick mobilization; no complications. Patient 5: time to union 50 weeks; went from in-house mobilization to hoist; no complications. The time frame or final follow-up period was not mentioned

Discussion

The present study aimed to summarize the currently available literature regarding postoperative weight-bearing regimes in patients with a surgically treated late PPFF after THA, with a special focus on PWB during aftercare. The main finding of this scoping review was that, after an extensive systematic search, no literature was available focusing on PWB aftercare in patients with a late PPFF after THA.

PWB in aftercare for late PPFF can offer several advantages, which are in line with studies reporting on PWB for other lower extremity fractures. First, it can lead to reduced complication rates [10,11]. PWB allows patients to start weight bearing on the affected limb earlier than traditional NWB protocols [13]. This can help reduce the risk of complications associated with prolonged postoperative immobilization, such as pneumonia and delirium [8,9]. Second, PWB promotes improved mobility and functional recovery [10,11]. Patients can regain independence more quickly, which can lead to a positive impact on their overall quality of life [14]. Third, controlled weight bearing can stimulate bone healing and remodeling by promoting physiological stress on the fracture site [25,26]. This may lead to faster bone healing outcomes. Fourth and last, PWB can therefore theoretically facilitate shorter hospital stays, reducing healthcare costs and the burden on both patients and healthcare systems.

Weight bearing is typically measured using percentages of body weight or categorized as NWB, RWB, and FWB. These methods can make it challenging for therapists to accurately gauge the amount of weight being applied at the fracture site during rehabilitation and daily activities [10]. Consequently, clinical practice may inadvertently involve excessive or insufficient weight bearing, potentially resulting in complications or prolonged recovery periods [10]. PWB entails gradually progressing the weight-bearing protocol by considering both subjective assessments made by the patient and therapist, including pain thresholds and the patient's capacity to endure weight bearing [13]. Additionally, objective factors such as limb temperature, swelling, and gait measurements are taken into careful consideration during the rehabilitation process [13].

The current literature search showed that PWB is not widely used as a postoperative aftercare regimen in surgically treated late PPFF. First and foremost, the lack of evidence-based PWB protocols in the literature underscores a prevailing trend toward more traditional weight-bearing regimens, such as NWB and RWB. These conventional approaches have been the norm in the management of PPFF. However, the limited inclusion and exploration of PWB in current literature suggest that this approach has not yet gained widespread recognition or acceptance. Also, in general, prescribed postoperative weight-bearing regimes were not always clearly described. Furthermore, the terminology used for weight-bearing regimens is not standardized across studies. In this review, we found multiple studies describing postoperative care regimes similar to PWB (e.g., weight bearing as tolerated), but not explicitly using the term PWB. Therefore, it is challenging to identify and assess studies that incorporate this aftercare regimen. This lack of consistent terminology contributes to an underrepresentation of PWB in the literature.

Further research is warranted to elucidate the optimal balance between early weight bearing and the risk of complications in this patient population. Clinical studies should aim to systematically investigate the efficacy and safety of PWB protocols, with a focus on defining and standardizing the terminology. By doing so, the involved practitioners can gain a more comprehensive understanding of the potential advantages and drawbacks of PWB, ultimately informing evidence-based practices and improving patient outcomes in the management of late PPFF around THA.

*Strengths* *and Limitations*

There are some strengths and limitations to this scoping review. Strengths of the present systematic scoping review, which centers on postoperative weight-bearing regimes in patients undergoing surgical treatment for late PPFF, include first that this review distinguishes itself as the inaugural study to comprehensively analyze and present findings concerning postoperative weight-bearing regimes in this patient category. It stands as a trailblazer in the field, shedding light on a crucial but previously underexplored aspect of late PPFF management. Second, we delved into the subject of postoperative weight bearing, with a particular emphasis on PWB protocols. By synthesizing existing literature, this scoping review provides an up-to-date overview of the knowledge in the field of postoperative weight-bearing regimes for surgically treated late PPFF within the scope of this review. Third, the comprehensive nature of this review and its emphasis on PWB may pave the way for the development of evidence-based guidelines for postoperative weight-bearing management in late PPFF. The focus on PWB is particularly valuable, as it addresses a critical aspect of postoperative care that can significantly impact patient outcomes. Such guidelines, which, to our knowledge, still do not exist, can improve the consistency and quality of care provided to patients in this category.

The first limitation was that there were only retrospective studies and case reports available in the included literature. Second, multiple studies clearly described their unique population, including the type of late PPFF and postoperative weight-bearing regime, but did not stratify results on the different types of fractures or based on the postoperative weight-bearing regime. For that reason, we were unable to extract relevant data from these studies. Third, because of the limited number of included studies, it is nearly impossible to draw valid conclusions with great certainty from the data obtained.

## Conclusions

The present systematic scoping review stands as a contribution to the field, offering a unique and valuable perspective on postoperative weight-bearing regimes in surgically treated late PPFF with a specific focus on PWB. Within the scope of this review, we were not able to include any literature concerning surgically treated late PPFFs focusing on PWB as an aftercare regimen. Therefore, we cannot conclude that PWB is a safe and viable option in this patient category. Addressing this gap in the literature is essential to advancing the understanding of postoperative weight-bearing protocols and PWB for late PPFF around THA.
